# The Preparation and After-Treatment of Abdominal Operations

**Published:** 1908-12-26

**Authors:** Arthur E. Giles

**Affiliations:** Surgeon to the Chelsea Hospital for Women; Gynæcologist to the Prince of Wales's General Hospital, Tottenham


### Hospital Clinics.

### THE PREPARATION AND AFTER-TREATMENT OF ABDOMINAL OPERATIONS.

By ARTHUR E. GILES, M.D., B.Sc. Lond., F. R.C.S. Edin.; Surgeon to the Chelsea Hospital for Women; Gynæcologist to the Prince of Wales's General Hospital, Tottenham.

### II.—AFTER-TREATMENT OF ABDOMINAL OPERATIONS.

(Delivered at the London Medical Graduates' College and Polyclinic, November 18, 1908.)

The treatment of patients after abdominal operations has changed a good deal during the last ten years; and it may prove useful if I give a short account of the main features of present-day treatment. If we contrast past and present we may summarise the more important points of divergence as follows: In the past, all food and drink were withheld for twenty-four hours; the patient was kept rigidly on her back for a week; morphia was frequently administered; aperients were given about the third or fourth day; and the patient was not allowed to sit up without putting on a cumbersome belt. At the present time, liquid nourishment is given within the first six or twelve hours; the patient is propped up in bed on the first or second day and allowed to lie on her side in a few days; morphia is given very rarely; an endeavour is made to secure an action of the bowels on the day after operation; and belts are discarded in all but exceptional cases. The older treatment was in many respects a great hardship for the patient; the twenty-four hours without a drink were a veritable purgatory, and patients have been driven in their desperation to resort to dangerous measures, such as seizing the opportunity, when the nurse's back was turned for a minute, to drink out of their hot-water bottles. This happened with two of my patients some years ago, and though neither suffered any ill-effects, it is naturally not a proceeding to be encouraged. Then, again, the position, flat on the back, was responsible for a very distressing amount of backache, as any one will appreciate who tries lying flat for even six hours. In other respects the older treatment was fraught with risk: in any case in which there was a danger of peritonitis, the two things that would most readily favour its development would be to give morphia and leave the bowels unopened for three days, and I have no doubt that a number of patients succumbed in consequence. An abdominal belt is unwieldy and uncomfortable, and by keeping the abdominal muscles out of active use leads to weakness of the abdominal wall. Thus the present-day patient is in many respects more fortunate than her predecessors.

I propose to review in brief first, the after-treatment of an uncomplicated abdominal case; and then the danger-signals and the treatment of complications.

#### After-Treatment in a Normal Case.

*The Return to Bed.*—During the operation the nurse has prepared the bed for the patient. The bed should be well warmed by means of hot-water bottles and these should be removed before the patient is put to bed. It is a useful working rule never to leave hot-water bottles in bed with an unconscious patient; and if, exceptionally, it is necessary to leave them, the patient must be carefully protected by blankets from contact with them and the resulting risk of burns. A pillow is placed under the knees; the head is left without a pillow, or with a very low one, and is turned on one side with a towel placed in such a way as to protect the patient and the bed in case of sudden sickness. A small bowl should be at hand, and a few cotton-wool mops for the purpose of clearing the throat of excess of mucus. It is observed that the secretion of mucus is much more free after ether administered through a Clover's inhaler: it is much less after chloroform and after ether given by the open method. A single blanket should cover the patient; over this is placed a cradle, covered in turn by the remaining bed-clothes.

*Early Treatment.*—For the first few hours the patient should not be left alone for a minute. Some patients are hysterical, and before the return to full consciousness they are liable to all kinds of dangerous pranks, including even getting out of bed; in other cases they want careful watching lest they should choke while vomiting or as the result of over-secretion of mucus, or lest syncope should supervene, when there is a tendency to shock. If shock be feared, a hypodermic syringe ready filled with a dose of strychnine or of ergotin may be left with the nurse to be used if necessary; but it is even better to anticipate such a contingency by the administration of an enema of three ounces of beef tea with an ounce of brandy; or, better still, by the rectal injection of two to three pints of normal saline solution, run in very slowly from a douche-can, only slightly raised above the patient's level. Such an injection has the additional advantage of diminishing the tendency to thirst.

Occasionally the patient is very restless, and the medical attendant may be tempted to give morphia. This is a temptation to which there must be no yielding. My most earnest admonition to any one who is going to look after a patient of mine is "no morphia as you love me!" A tactful and attentive nurse can almost always restrain a patient from throwing herself about until such time as she is sufficiently conscious to listen to reason.

During the first few hours the patient's mouth should be washed out frequently with hot water; a few drops of sal-volatile or brandy added thereto will help to remove the nauseating taste of the anæsthetic. There is no harm in allowing the patient to swallow a little, for even should she be sick the water will help to rid the stomach of the bile and ether-impregnated mucus whose presence encourages nausea and retching.

If the patient appears inclined to sleep, even for a considerable time after an operation, no attempt should be made to rouse her; sleep is quite her best friend during the early miserable hours.

*Sedatives and Hypnotics.*—When pain is severe, we have to look for some substitute for morphia. This, as I have said, is contra-indicated, and the reason is that the bowels cannot be allowed to sink into torpor; they must be kept moving on, otherwise they undergo a passive distension, which, at the best, causes pain and discomfort from flatulence, which may be worse and more troublesome than the initial pain, and, at the worst, leads to intestinal obstruction and favours sepsis. The best substitutes for morphia are ammonol, phenacetin, and chloretone in 10-grain doses, given preferably as a powder in a cachet, or 20 grains each of ammonium bromide and chloral may be given by the rectum. If the patient is wakeful and restless, but has no particular pain, we may try veronal, trional, or aspirin, in 10-grain doses, repeated, if necessary, in three or four hours. When want of sleep goes beyond the first day or two, a mixture may be given three times a day with advantage, containing 10 grains of ammonium bromide, 15 minims of compound tincture of cinchona, and 1 drachm of syrup of codeia to the dose.

*Posture.*—It is by no means necessary to keep a patient lying flat on her back for days; even with a knee-pillow the strain on the back is great, and induces a very distressing form of backache. It is permissible to turn her on her side on the day after the operation; she will probably find that after twenty minutes or half an hour she is glad to lie on her back again, but meanwhile her muscles have had the rest that comes from change of position. The patient must, of course, be impressed with the fact that her conjugation of the verb "to move" must be entirely in the passive mood. The treatment of septic peritonitis by means of Fowler's position has given us an indication for dealing with normal cases. In this position the patient is made to sit up almost vertically with a bed-rest as soon as she recovers consciousness; and to prevent her from slipping down straps or bandages are fastened to the ends of the knee-pillow, and their other ends are fixed to the head of the bed in such a way that she sits in a kind of sling. Now, if septic patients can assume this position with comfort, there is no reason why uncomplicated cases should not be similarly treated, and as a matter of experience we find that patients get great relief from this posture; they are able to sleep, they show less tendency to sickness, and the backache is almost entirely done away with. I have been adopting this position as a matter of routine after abdominal operations, and have been very pleased with the results. It is not, of course, necessary or desirable in an ordinary case to keep a patient constantly sitting up; she may be allowed to assume the horizontal position from time to time, and especially at night. During the day, and particularly for meals, sitting up is a great relief; and when such a patient begins to get up she is stronger, and shows less tendency to faintness, than one who has been kept recumbent the whole time; naturally, with a very anæmic patient, or with one who is suffering from shock, the sitting-up posture is contra-indicated.

*Food.*—A certain amount of discretion must be exercised in the question of nourishment after operation, as a good deal depends on whether the patient is sick or not. In the absence of sickness there is no necessity for the long period of starvation that used to be enforced. Drinks of hot water may be allowed within a few hours, and six to eight hours after the operation a small drink of milk tea can be given. If this is retained and the patient does not feel sick, milk and barley water in equal parts are given in gradually increasing quantities every hour, varied with drinks of milk-tea or milky coffee, cocoa, beef-tea, Benger's food, cornflour, or milky gruel. A little Brand's jelly is often enjoyed. This type of nourishment is kept up for 48 hours. Then, when the bowels have acted, solid food is begun, in the form of bread and butter with the tea, a lightly boiled egg, and custard. About the fourth day we may give a purée of fish, minced meat, or boiled sole or plaice; chicken, sweet-bread, fried sole, or game, with milk puddings, then forms the diet for the remainder of the first week. From the second week onwards the diet gradually assumes normal proportions, vegetables and stewed fruits being added. When a patient shows a continued tendency to sickness, food by the mouth is often best withheld for 24 hours, and a nutrient enema consisting of 4 oz. of peptonised milk and 2 drachms of meat-juice is given every four hours. I very seldom order stimulants after operation, but I

PRINCE OF WALES'S GENERAL HOSPITAL, TOTTENHAM.

After Treatment of an Abdominal Operation.

| Within 24 Hours (Operation Day, Thursday) | First Day (Friday) | Second Day (Saturday) | Third Day (Sunday) | Fourth Day (Monday) | Seventh Day (Thursday) |
|---|---|---|---|---|---|
| *Diet.*<br>No food while sickness lasts.<br>ʒj. milk and water, soda or barley water (according to taste) given hourly, beginning 8 hours after operation.<br>Small quantity of freshly-made tea if doctor allows. (In most cases there is some sickness, and nourishment is delayed until 16 to 18 hours after operation).<br>At the end of 12 hours, if absolutely necessary, the catheter must be passed, to be repeated 8-hourly for 48 hours (perineorraphy), or until patient is able to pass urine naturally. | *Diet*<br>ʒv. milk, 2 hourly, varied with beef-tea, coffee, or tea.<br><br>Patient is propped up in a sitting position, supported by straps.<br>If troubled with flatulence and distension an enema terebinth may be given. | *Diet.*<br>Benger's food, cornflour, arrowroot, etc.<br>Milk, broth, beef-tea, coffee, etc.<br><br>An aperient is given in the evening. Either:—Ol. ricini, ʒi.; or mist. cas., ʒi. | *Diet.*<br>Custard and jelly, milk, small quantities of bread and butter, bread and milk, etc.<br>In the morning: an enema saponis. | *Diet.*<br>Milk pudding, custard, lightly-boiled eggs, jelly, milk, broth, beef-tea, etc., until seventh day.<br>An aperient at night if necessary. | *Diet.*<br>Boiled fish, chicken-mince, milk puddings, milk, beef-tea broths, etc.<br><br>Stitches removed. Four-hourly chart discontinued if condition of patient allows. |

occasionally allow them if the patient has been accustomed to them and requires some incentive to appetite. Without any prepossession against alcoholic compounds, I believe that they are rarely necessary, and that they may be advantageously replaced by hot milk.

In hospital practice, where it is necessary to have a routine plan applicable to the majority of cases, such plans as those adopted at the Chelsea Hospital for Women, and the Prince of Wales's General Hospital, Tottenham, may be carried out with advantage. They err, if anything, on the Spartan side. Miss Howard, the sister-in-charge of my wards at Chelsea, has kindly drawn up for me a time-table, based on the fact that my patients are operated upon on Monday afternoons. It shows the routine plan which is followed in an ordinary case. Miss Simmonds, Sister of the Louise (gynæcological) ward at the Prince of Wales's General Hospital, Tottenham, has also kindly made a schedule of the plan which is carried out in a normal case under her charge. I append both these plans, as they may serve as a useful guide in abdominal cases. I may add that the treatment appears to me to be justified by results. During the last five years I have had 254 abdominal operations at Chelsea, with six deaths, and in my last 250 cases at Tottenham there were eight deaths, giving a mortality of 2.8 per cent. for 500 cases.

### CHELSEA HOSPITAL FOR WOMEN.

Operation, Monday 2 p.m.

| | | | | |
|---|---|---|---|---|
| 7.30 | P.M. | Catheter passed. | | |

Tuesday.

| | | | | |
|---|---|---|---|---|
| 2.0 | A.M. | Nutrient enema. Pep. milk ʒiv. *c.* Brand's meat juice ʒij. Catheter and rectal tube. | | |
| 6.0 | A.M. | Tea (no sugar). | | |
| 9.0 | A.M. | Hot water ℥iij. | | |
| 10.0 | A.M. | Milk and barley water ... ... | aa. | ʒij. |
| 11.0 | A.M. | ,, ,, ,, ... ... | aa. | ʒiij. |
| 12.0 | A.M. | ,, ,, ,, ... ... | aa. | ʒiv. |
| 1.0 | P.M. | ,, ,, ,, ... ... | aa. | ʒv. |
| 2.0 | P.M. | ,, ,, ,, ... ... | aa. | ʒvj. |
| 3.0 | P.M. | ,, ,, ,, ... ... | aa. | ʒvij. |
| 4.0 | P.M. | Tea ℥iij. (no sugar). | | |
| 5.0 | P.M. | Milk and barley water ... ... | aa. | ℥j. |
| 6.0 | P.M. | ,, ,, ,, ... ... | aa. | ℥j. |
| 7.0 | P.M. | ,, ,, ,, ... ... | aa. | ℥j. |
| 8.0 | P.M. | ,, ,, ,, ... ... | aa. | ℥j. |
| 9.0 | P.M. | ,, ,, ,, ... ... | aa. | ℥ij. |

Drinks gradually increased through the night until ℥v. two hourly are reach by 8 A.M. Wednesday.

Wednesday.

Patient kept on fluid diet, not less than ℥v. two hourly (beef-tea, milk, cocoa). Brand's jelly in the morning and small piece of bread and butter without crust given with tea at 4 P.M.

Thursday.

Boiled sole or plaice and custard given for dinner.

Saturday and Sunday.

Fried sole or plaice and custard for dinner.

Monday.

Chicken or boiled mutton on alternate days for a week, with milk pudding.

---

In hysterectomy cases, and in other cases when necessary, the catheter is passed six-hourly for the first twenty-four hours.

In all abdominal cases the rectal tube is passed four-hourly unless the patient is passing flatus naturally.

A turpentine enema (turpentine ʒij., olive oil ʒiv., sterilised soap ʒiv. *c.* water oij.) is given on Wednesday morning.

An aperient is given on Wednesday evening, very often now on Tuesday evening.

The pulse, temperature, and respiration are recorded four-hourly.

The patient is propped up in the sitting posture from the second day onwards. On the eighth day the sutures are removed; on the fifteenth she is allowed to sit up in a chair and dressings are discontinued; on the nineteenth day she goes to the Convalescent Home at St. Leonard's or to her own home.

*Attention to the Bladder and Bowels.*—In most cases, when a patient is able to pass water naturally, she should be allowed to do so. If she is not able, the catheter must be passed at intervals not exceeding eight hours. Sometimes a patient is not able to relieve herself naturally for some days and even (as I have known in a few instances) for a week or two. If a patient seems to be hysterical or "faddy," she must be humoured. I have in mind the case of a patient who was told that her inability to pass water was all nonsense, and that she must strain till she managed it. She was not wanting in willingness, and she strained to such purpose that she burst open her wound, with protrusion of the bowel and the subsequent development of a fæcal fistula. On the other hand, some patients require to pass water frequently; it is the refinement of cruelty in such a case to insist on a bed-pan drill at fixed hours, and the nurse should be given to understand quite clearly that the patient's requirements in this respect must now be complied with, however exacting or even capricious she may appear.

With regard to the bowels, it is always advisable and often essential that they should be opened early. I advocate a dose of castor oil 24 to 36 hours after an operation, followed in a few hours by an enema. If distension is causing distress, and is not relieved by the passing of a rectal tube, a turpentine enema may be given within 24 hours. For the remainder of the time when the patient is in bed she will probably require an aperient at intervals; syrup of figs, senna pods, liquorice powder, aloin or cascara may be given, with a dose of calomel if necessary, and an occasional enema. Sometimes salines answer very well, given in repeated doses. In cases of sickness or of threatened sepsis an excellent plan is the administration of calomel in small doses of one-sixth to one-half of a grain every hour for six doses, or until a result is obtained.

*The pulse, temperature, and respiration* should be recorded every four hours for the first week, and twice a day afterwards, because they give valuable indications as to the patient's progress.

*The Dressing.*—As a rule the dressing may be left untouched for a week; all that is necessary is to adjust the binder from time to time. When this becomes loose, as it does when a patient who has been troubled with distension has been relieved by an enema, it should be tightened. It is important that the nurse should see that the dressings do not work up in such a way as to leave the lower part of the wound uncovered; if this happens the wound is liable to become infected. To prevent such an accident the best plan is to have a perineal bandage attached back and front to the binder; a pad of wool between the bandage and the patient's skin will prevent chafing.

On the eighth day the wound is dressed and the stitches are removed; fresh gauze and wool are placed over the wound, and the binder is re-applied. A few days later a simple collodion gauze dressing may be put on, and by the end of the second or third week all dressings may be omitted.

A surgical belt is hardly ever necessary, especially when the three-layer method has been used for securing the wound. A belt is heavy and cumbersome, and discourages the abdominal muscles from recovering their normal tone. When patients feel that they would like a little support for the first few weeks, I advise them to wear a woven or knitted woollen garment of the type known as a cholera or Jaeger belt.

*Massage.*—Many patients derive great benefit from massage. It may be begun on the limbs and chest a week after operation. At the end of the second week it is extended to the abdomen. When these patients get up they are markedly stronger than those who have had no massage.

*Getting Up.*—Patients should be kept in bed for not less than two weeks after an operation. This is the regulation time for my hospital patients. In other cases three weeks may be allowed, or longer if special circumstances require it. The first day the patient is transferred to a couch for an hour or two; on the next day she may sit up on the couch with her feet down, and try walking a little. The day after she can sit on a chair, and walk about rather more. When she has been up for four or five days she may go downstairs or go out for a short drive. At the end of a month from the time of operation she is well enough to leave the home and go away for a change.

*Later Convalescence.*—A careful medium course must be followed between letting a patient do too much or get about too soon and allowing her to remain an invalid; and a good deal depends on the attitude of the medical attendant. Some patients are encouraged to think themselves invalids for months and even years, and this is a mistake. After any ordinary uncomplicated abdominal operation the patient should quite cease to be an invalid at the end of three months, and should be able to resume ordinary avocations with only this reservation, that she should not undergo strenuous and fatiguing exercise, such as tennis and cycling, for yet another three months. But walking may be freely allowed, and I have known a patient resume golf with comfort and pleasure four months after an abdominal hysterectomy for fibroids.

### Danger Signals.

There are several important signs which serve as warnings that all is not going well.

*The Temperature.*—A rise of temperature to 100° or 101° F. within the first 24 or 36 hours need cause no anxiety; it is often a reaction from shock, and it is also found after an operation in which there have been signs of commencing inflammatory trouble. If the temperature rises rapidly to 103° or 104° F. look out for peritonitis. A continued temperature about 100° going on during the first week should lead to examination of the wound, as it may be due to superficial suppuration. A continued temperature of 101° or 102° may mean an inflammatory focus in the cellular tissue of the pelvis.

*The Pulse.*—As long as the pulse keeps down to 80 the outlook is generally good. A pulse of 100 to 120, full and bounding, is a sign of inflammatory trouble and accompanies the early stages of sepsis. A weak pulse about 100 to 120 is found with shock; and a weak-running pulse of 130 to 160 nearly always means active hæmorrhage. An irregular, intermittent pulse is a bad sign and calls for extra caution in allowing the patient up, as it may indicate a fatty heart. A weak, slow pulse is one that is calling aloud for more nourishment.

*Respiration.*—In a normal case the breathing should not be quicker than 20 to 24 per minute. Any acceleration over this should make us look out for bronchial and pneumonic complications. A sighing, rapid respiration with rapid pulse, restlessness and great pallor points to hæmorrhage.

*The Tongue.*—As long as the tongue remains moist and clean there is little danger: a dry brown tongue is a disconcerting sign, as it is often found in the early stages of sepsis, and if it becomes hard, coated, and dark brown or black the outlook is very bad: such patients often die.

*Abdominal Distension* is not infrequent during the first 48 hours, and if flatus is being passed no anxiety need be felt. Nevertheless, I like to feel a hollow in the epigastrium between the ribs. When no flatus is being passed by the end of the second or third day we must look out for sepsis (with a rising temperature) or intestinal obstruction (with a normal or falling temperature and vomiting).

*Vomiting.*—Many patients are sick for a few hours after operation: the vomiting becomes alarming only when it persists into the second or third day, and more particularly if it becomes fæcal. "Brown vomit" is also a very bad sign: it is found both with sepsis and with commencing obstruction. Persistent vomiting may also be a sign of acetonæmia.

*Suppression of Urine* is generally a grave sign. It may be an indication of nephritis, or it may be the result of some operative accident such as injury to bladder or ureters. We must be careful to distinguish between suppression and retention; the passing of a catheter will at once settle the question.

### The Treatment of Complications.

It will be convenient to consider first the slighter complications and then those that are more serious.

*Shock.*—This is best met by a rectal injection of normal salt solution or a small nutrient enema of brandy (1 oz.) and beef tea (3 oz.). At the same time the patient should be kept warm with hot-water bottles, and the foot of the bed may be raised.

*Sickness.*—If sickness continues beyond the first hour or two, two teaspoonfuls of bicarbonate of soda may be given in a feederful of hot water. This will probably be returned, washing out the stomach at the same time; a further teaspoonful is then given in 2 oz. of hot water, and this will probably be retained and the sickness will cease.

If there is still some sickness, a drop of tincture of iodine in 1 oz. of water often acts like a charm. Sometimes the feeling of nausea is lessened by propping the patient up in bed. In obstinate cases of sickness all nourishment by the mouth should be withheld for twenty-four hours, and a nutrient enema should be administered four-hourly, consisting of four ounces of peptonised milk and two drachms of meat-juice.

*Flatulence* is often rather troublesome, and sometimes it is difficult to relieve during the first twenty-four hours. A rectal tube passed four-hourly will sometimes facilitate the expulsion of wind. If it persists, a turpentine enema should be given. The formula of the Chelsea Hospital, given above, is a useful one, one to two pints being injected.

*Bronchitis.*—In a patient predisposed to chest trouble we may presume that chloroform has been given instead of ether; but if, nevertheless, bronchial symptoms appear, an expectorant mixture should be given, and a bronchitis kettle, with compound tincture of benzoin, may be requisitioned.

*Hæmorrhage.*—When symptoms of secondary hæmorrhage come on, as indicated by marked pallor, a running thready pulse, restlessness, and sighing respiration, no time should be lost, but preparations for reopening the abdomen should be made at once. Meanwhile the foot of the bed should be raised, and the patient should be kept as quiet as possible. No stimulants should be given. As soon as the bleeding-point has been secured, a venous saline transfusion should be resorted to.

*Intestinal Obstruction.*—When this threatens, an effort should be made at once to secure an action of the bowels by the administration of calomel in divided doses, followed by repeated doses of sulphate of soda and a copious enema. Expectant treatment must not be carried on too long, however; and if fæcal vomiting comes on, the abdomen should be reopened as soon as possible.

*Sepsis.*—When once a case becomes septic the outlook is exceedingly grave; nevertheless, we must not fold our hands and leave the patient to her fate. The principles of treatment here are abdominal drainage, the administration of large quantities of saline solution *via* the rectum, and the propping up of the patient in the sitting-up posture, in order to protect the vulnerable diaphragmatic area of the peritoneum as much as possible. Calomel should be given early, in divided doses, and an injection of some anti-toxic serum may be tried, according to the nature of the case. Every effort must be made to maintain the patient's strength, and large quantities of stimulant are called for.

*White-leg* is generally due to a more or less septic thrombosis of the veins and lymphatics. The patient should be kept absolutely at rest, with the affected limb slightly raised and enveloped in wool; belladonna and glycerine may be painted over the affected area. Gentle uniform pressure should be applied, but not by the usual roll-bandage, whose application disturbs the leg too much; a sheet of calico should be passed under the limb and fastened down the front with safety-pins.

*Parotitis* should be treated by fomentations, and by incision if a definite abscess forms.

*Pulmonary Embolism* is the worst of all complications to face, because no treatment is of any avail. The inhalation of oxygen offers the only possible chance.

